# A Comprehensive Analysis of Luminescent Crystallized Cu Nanoclusters

**DOI:** 10.1021/acs.jpclett.3c03374

**Published:** 2024-01-22

**Authors:** Sourav Biswas, Yuichi Negishi

**Affiliations:** † Department of Applied Chemistry, Faculty of Science, Tokyo University of Science, 1-3 Kagurazaka, Shinjuku-ku, Tokyo 162-8601, Japan; ‡ Research Institute for Science & Technology, Tokyo University of Science, 1-3 Kagurazaka, Shinjuku-ku, Tokyo 162-8601, Japan

## Abstract

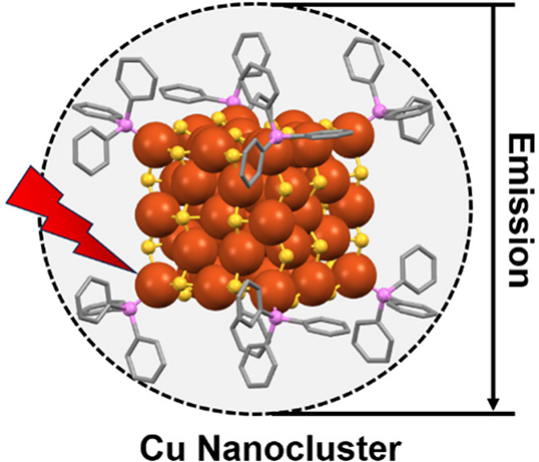

Photoluminescence (PL) emission is an intriguing characteristic displayed by atomically precise d^10^ metal nanoclusters (NCs), renowned for their meticulous atomic arrangements, which have captivated the scientific community. Cu(I) NCs are a focal point in extensive research due to their abundance, cost-effectiveness, and unique luminescent attributes. Despite similar core sizes, their luminescent characteristics vary, influenced by multiple factors. Progress hinges on synthesizing new NCs and modifying existing ones, with postsynthetic alterations impacting emission properties. The rapid advancements in this field pose challenges in discerning essential points for excelling amidst competition with other d^10^ NCs. This Perspective explores the intricate origins of PL emission in Cu(I) NCs, providing a comprehensive review of their correlated structural architectures. Understanding the mechanistic origin of PL emission in each cluster is crucial for correlating diverse characteristics, contributing to a deeper comprehension from both fundamental and applied scientific perspectives.

The field of metal nanocluster (NC) research is experiencing remarkable progress as researchers delve into the intricacies of diverse structural architectures and their associated properties.^1−5^ One intriguing aspect of this research is the transition from traditional metal candidates like gold (Au) and silver (Ag) to copper (Cu).^6−17^ Similar to other NCs, Cu NCs also possess a noteworthy quality known as monodispersity, which is characterized by well-defined molecular formulas.^18^ However, a significant challenge in this domain is achieving that precise molecular purity at the nanoscale.^19^ This challenge is rooted in the elusive growth mechanisms that govern the formation of all of the other NCs. Recent scientific endeavors have shed light on certain key factors that offer precise control over the growth of general NCs.^12,20−22^ Importantly, these factors are intricately tied to the choice of metal atom used in the NC formation process. As researchers make the transition to Cu from their more traditional Au and Ag counterparts, they encounter challenges analogous to those faced during the initial exploration of NCs.

Beyond the synthesis of NCs, ensuring their stability is of paramount significance.^23−25^ Cu, despite sharing the same group on the periodic table with Au and Ag, exhibits a notably distinct half-cell reduction potential. This differential potential significantly influences the stability and the associated properties of the newly synthesized NCs.^26−28^ Furthermore, the structural architecture of ligands, molecules, or ions that attach to the surface of the NCs assumes a pivotal role in shaping the overall stability of these NCs.^29,30^ Ligands act as a protective shield, forming a crucial layer that separates the metallic core of the NC from the external environment.^12,31^ This barrier contributes significantly to the structural integrity of the NC.^32,33^ Interestingly, in the traditional noble metal NCs, it has been observed that the size and conformation of these protective ligands also influence the precise properties of the NCs.

The captivating property of photoluminescence (PL) distinguishes itself prominently in the realm of atomically precise d^10^ metal clusters.^34^ The luminescent characteristics of these clusters are rooted in the charge transfer phenomenon primarily shaped by metallophilic interactions and ligand properties. Particularly, the focus is on Cu(I) clusters within this class, garnering considerable attention due to their abundance, cost-effectiveness, versatility in reacting with various ligands, and favorable low-energy ligand-to-metal charge transfer or cluster-centered transitions. These specific attributes open up exciting possibilities for diverse applications, such as the advancement of highly luminescent sensors, the creation of innovative biomarkers, and the design of organic light-emitting devices with enhanced luminosity.^19,35−37^ However, due to size constraints, these NCs lack characteristics of surface plasmonic resonance at 500–600 nm.^38^ Instead, there is a prevalent emergence of absorption peaks in the higher wavelength region. These peaks are often attributed to interband electronic transitions, and their characteristics are inclined to the protective ligand system.^39,40^ Siwach and coauthors meticulously investigated the luminescent characteristics of Cu NPs.^41^ Their comprehensive study unveiled a weak PL emission with a peak at 296 nm. This emission phenomenon was ascribed to transitions originating from excited states to Cu 3d. Concurrently, an earlier investigation conducted by Vázquez et al. reported Cu NCs synthesis through a microemulsion technique.^42^ In this instance, the observed PL emission was exhibited in a distinct blue region. However, subsequent research endeavors have brought to light a spectrum of PL emission colors associated with different Cu NCs, all over the visible region.

Initially, the assumption prevailed that discrepancies in emission properties between NPs and NCs were confined to their quantized electronic orbitals.^43^ To substantiate this notion, the Jellium model was introduced, with the intention of elucidating the emission characteristics of NCs across varying size scales. This model proved effective at starting in elucidating the emission properties of diverse Cu NCs. For instance, Cu NCs with metal cores of Cu_5_, Cu_13_, and Cu_20_, stabilized by [N(C_4_H_9_)_4_]^+^, exhibited PL emissions at 305, 425, and 500 nm, respectively.^44^ However, this apparent simplicity was short-lived, as exceptions emerged within this series. Cu_13_ NCs stabilized by BSA (bovine serum albumin), for instance, defied the trend by displaying PL emission peaking at 410 nm.^45^ Another deviation was observed in histidine-protected Cu NCs, which contained varying numbers of Cu atoms, mostly ranging from 3 to 6, yet exhibited nearly identical emission maxima around the 456 nm region.^46^ Consequently, the Jellium model fell short in accurately describing the emission properties as the number of Cu NCs increased, particularly those with surfaces protected by diverse ligands. This underscores the key role of ligands. Delving into the intricate relationship between surface ligands and the emission properties of Cu NCs necessitated the acquisition of structurally characterized Cu NCs. This pursuit embarked upon a protracted journey, tracing its origins back to the synthesis of copper-halide complexes. The synthesis of these Cu(I)-halide complexes has been a focal point of researchers, primarily due to their remarkable emissive properties and high quantum yields (QYs).^47−51^ The majority of these Cu(I)-halide complexes have garnered attention for their pronounced phosphorescence, typically manifesting in the 550–650 nm range.^52,53^ Moreover, in the solid state these complexes exhibit intriguing thermochromic behavior.^53^ This behavior can be understood through the interaction between the cluster-centered triplet and the dynamics of charge transfer that occurs between the halide ligands and the Cu atoms within the complexes. This intricate interplay exposes the detailed mechanisms that govern the emission characteristics of these complexes, underscoring the significance of a comprehensive understanding of the structural distinctions inherent in them.

Subsequent to a dedicated exploration for novel Cu NCs featuring distinct structures, a plethora of organic ligands, such as thiolates, phosphines, and alkynes, have been systematically employed.^11,28,54−65^ However, despite these endeavors, not all of these NCs exhibit emission characteristics. In this context, our discussion delves into the subset of structurally characterized Cu NCs that do demonstrate luminescence properties, as we strive to unravel the underlying origins of their luminescent behavior. Langer et al. synthesized and crystallized a series of Cu(I)-thiolate complexes and examined their PL emission properties.^66^ While employing a reaction protocol similar to the one used previously, they employed distinct tertiary mono- or bidentate phosphane ligands. However, they observed no direct connectivity between Cu(I) atoms, but they identified the bond distances lower than twice the van der Waal radius of Cu(I) atom in some of the cases. So, based on this consideration we choose two polynuclear Cu(I) complexes which can be assigned as Cu NCs which are [Cu_7_(*p*-S-C_6_H_4_-NMe_2_)_7_(PPh_3_)_4_] (Me: methyl; Ph: phenyl) (Figure 1a) and [Cu_7_(*p*-S-C_6_H_4_-OSiMe_3_)(SPh)_6_(PPh_3_)_4_] (Figure 1b). Both of these NCs were synthesized using a uniform reaction protocol, with the only variation being the thiolate ligands employed. Despite crystallizing in the same space group, signifying a comparable geometric architecture, the average cuprophilic interactions differ due to the distinct coordination thiolate ligands. Specifically, the Cu–Cu distance in the NC with a single thiolate ligand is 2.7871, while in the NC with mixed thiolate ligands it is 2.8201. Although both NCs exhibit similar solid-state absorbance characteristics, disparities emerge in their absorbance edges. The detected absorptions predominantly result from charge transfer, influenced by cuprophilic interactions. The variation in absorption wavelengths is attributed to the favored electron donation ability from dimethylamine groups compared to trimethylsiloxyl substituents. This observation aligns with the solid-state emission properties, where the mixed thiolate-ligand-containing Cu NC displays a 30 nm blue shift in the emission maximum, reaching 530 nm.

**Figure 1:**
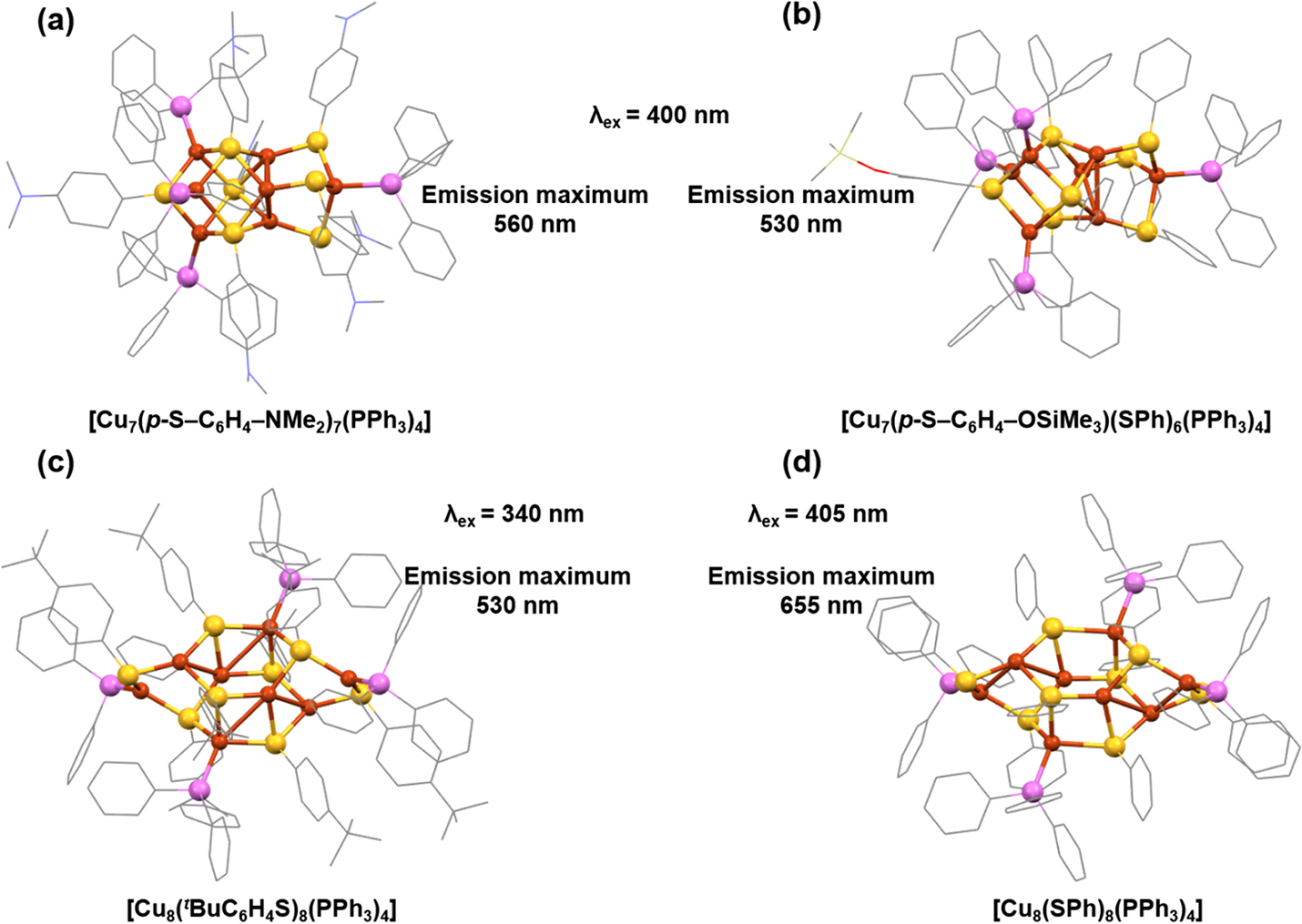
Structural architecture of (a) [Cu_7_(*p*-S-C_6_H_4_-NMe_2_)_7_(PPh_3_)_4_], (b) Cu_7_(*p*-S-C_6_H_4_-OSiMe_3_)(SPh)_6_(PPh_3_)_4_], (c) [Cu_8_(^*t*^BuC_6_H_4_S)_8_(PPh_3_)_4_], and (d) [Cu_8_(SPh)_8_(PPh_3_)_4_] NCs and their difference in emission maximum with different excitation. Recreated from the cif deposition of the original articles. In all structures, Cu is displayed in brown, S in yellow, P in violet, O in red, N in blue, Cl in green, and C in gray sticks. Other ligands were removed for clarity.

The ligand serves as a pivotal factor influencing alterations in cluster properties as it is mostly dependent on their structural architecture. Any change in the ligands part will directly influence the overall architecture of the cluster.^67^ In addition, the affinity of the cluster for a specific solvent is significantly contingent upon the nature of the ligand. Furthermore, the stability of the cluster hinges on the effectiveness of the ligands in shielding a delicate core from the surrounding environment. This underscores the intricate role of ligands in not only dictating solvent compatibility but also safeguarding the stability of the cluster against external influences. Sun et al. successfully synthesized and crystallized [Cu_8_(^*t*^BuC_6_H_4_S)_8_(PPh_3_)_4_] (Bu: butyl) NC (Figure 1c), revealing a distinctive metallic architecture resembling a twisted Cu_6_ octahedron.^68^ Two additional Cu atoms cap opposite triangles through weak cuprophilic interactions, creating a structural arrangement unique to the previous. The measured Cu–Cu distances were 2.796(9) to 3.024(8) Å. The protective ligands are strategically affixed to the surface of the NC, promoting favorable intercluster π–π interactions that drive the supramolecular assembly of the cluster. This supramolecular assembly not only optimizes bond distances between the Cu atoms but also enhances the interaction between them. In a dichloromethane solution, it displays an absorbance peak at 262 nm, closely corresponding to the calculated peak at 271 nm. Theoretical calculations suggest that this absorption band originates from the transition between the HOMO–9 and LUMO+2 orbitals, wherein the occupied orbitals are predominantly composed of Cu and S orbitals and the unoccupied orbitals consists mainly of PPh_3_ ligands. So, this orbital distribution suggests the presence of a metal-to-ligand charge transition in this context. Upon excitation at 340 nm, the NC demonstrates solid-state emission, peaking at 530 nm with a QY of 8.74%, and an emission lifetime of 8.36 μs. Interestingly, it was observed that the emission energy remains unaffected by temperature changes, suggesting restricted intramolecular rotation in its solid state. The high-energy emission band with a large lifetime is likely to be attributed to a mixed charge-transfer phenomenon. However, to compare the PL emission properties of this supramolecular self-assembly with the kinetically controlled supramolecular assembly, they prepared thermodynamically stable aggregate of this NC in dichloromethane and ethanol medium. They identified that the thermodynamically stable aggregate exhibits a maximum emission peak at 660 nm, indicating a lower emission energy compared with what is observed in the solid state. This variance is ascribed to the prevalence of the cluster-centered transition state within the thermodynamically stable aggregate. So, the low-energy emission band emerges from the cluster-centered transition process, distinct from the mixed charge-transfer mechanism observed in the solid state of the same NC. They also uncovered that the cluster-centered transition state is profoundly influenced by the solvent polarity. This influence plays a pivotal role in controlling the degree of aggregation, thereby regulating the cuprophilic interactions within the cluster unit. This intricate interplay enhances the intensity of the cluster-centered transition and concurrently reduces its emission energy, highlighting the intricate relationship between solvent conditions and the photophysical properties of the thermodynamically stable aggregate. The impact becomes more prominent in Cu_18_H(PhC_2_H_4_S)_14_(PPh_3_)_6_(NCS)_3_ NC, featuring a pseudo *D*_3_-symmetrical triple-helical Cu_15_ core.^69^ Distinctive interactions enable this nanocomposite to demonstrate enhancements in emission due to crystallization and aggregation effects in the deep-red spectrum. The intriguing influence of ligands on cluster sizes, coupled with the capacity of altering reaction conditions to stabilize clusters of similar sizes, opens up a valuable avenue for exploring and understanding the characteristics of clusters sharing identical core sizes but featuring diverse ligands. This unique capability provides an exceptional opportunity to delve into the intricacies of cluster–ligand interactions and their consequential effects on properties. For example, Ke et al. reported another Cu_8_ NC with a different thiolate ligand and denoted by the formula [Cu_8_(SPh)_8_(PPh_3_)_4_] (Figure 1d).^70^ The synthesis involved a one-pot reaction process wherein CuCl was treated in the presence of thiolate and phosphonate ligand precursors in an acetonitrile and dichloromethane solution mixture, followed by NaBH_4_ reduction. The resulting molecular architecture features two Cu_4_S_4_P_2_ units intricately linked by four S–Cu bonds. A notable aspect is the dual coordination modes exhibited by S atoms within the Cu_8_ cluster, and the presence of a cuprophilic interaction at the cluster node adds an additional layer of complexity. Delving into the investigation of luminescent properties in the solid state, they observed an emission band with its peak centered at 655 nm. Upon cooling the system to 80 K, a noticeable 35 nm shift was observed in the maximum emission band, accompanied by the appearance of a new peak at 560 nm. So, this NC displays a dual-emission profile in the solid state at 80 K. This unique phenomenon was ascribed to a synergistic interplay between the cluster-centered excited state and metal–ligand charge transfer, triggered by the temperature-dependent Cu–Cu distance. Hence, the noticeable disparities in the emission properties observed in the two Cu_8_ NCs can be can be traced back to the varied metallic arrangements facilitated by diverse ligand compositions. As integral components of the NCs, variations in ligands directly influence the cuprophilic interactions within the solid-state environment. Thus, these variances in cuprophilic interactions contribute to the diverse manifestation of emission characteristics in the Cu NCs, highlighting the intricate relationship among ligand composition, metallic arrangement, and resulting optical properties in the solid state.

Observations indicate a shift in emission properties from Cu_7_ to Cu_8_ NCs, leading to the conclusion that the emission characteristics of Cu NCs are intricately linked to the number of Cu atoms in the structure, determining the size of the NC as well. Hence, the modification of the Cu atom count is essential, and this can be accomplished by making changes to the ligand architecture given its significant influence on the inner structure. While phosphonate ligands are commonly employed to ensure the stability of a specific structure, their impact on determining the overall number of Cu atoms in a structure is limited. Therefore, the scope for modifying the core structure narrows down, with thiolate ligands emerging as the primary option for effecting changes in the Cu atom composition within the thiolate of a mixed ligand containing Cu NCs. Adhering to this procedure, Li et al. synthesized the [Cu_11_(SC_10_H_13_)_9_(PPh_3_)_6_]^2+^ NC (Figure 2a) by using 4-*tert*-butylbenzenethiol.^71^ The Cu(I) complexes were produced by treating CuCl with ligand precursors, in conjunction with tetraoctylammonium bromide, in a chloroform and methanol solution mixture. Subsequent reduction was achieved by using NaBH_4_. The complex structure of this NC reveals a triangular bipyramidal Cu_5_ core unit encircled by three Cu_2_S_3_P_2_ motifs. Remarkably, the distances between the metal atoms within the Cu_5_ unit span from 2.783 to 3.295 Å, indicating weak cuprophilic interactions. In a chloroform medium, this NC displays a UV–vis spectrum with a shoulder peak appearing at 400 nm (Figure 2b). However, an increase in the shoulder peak intensity was observed at low temperature without any noticeable shifts. They attributed this observation to elucidating the stability in the electronic structure of this NC at lower temperatures. Additionally, they determined that this transition corresponds to the HOMO–3 to LUMO+16 transition. The Kohn–Sham molecular orbital analysis reveals that occupied orbitals consist of Cu(3d) and S(3p) orbitals, while unoccupied orbitals are composed of the surface ligands. In addition, the chloroform solution of this NC displays an emission maximum at 685 nm, corresponding to the transition from the core to the ligands upon excitation at 400 nm (Figure 2c). However, the solid-state emission peak undergoes a noticeable blue shift of ∼10 nm compared with the solution. The absolute QY of the solid-state emission is 22%, significantly higher than the QY in the chloroform solution, which is 7%. This difference is attributed to the confinement of intramolecular motions in the solid state. This observation was further confirmed by examining the emission spectrum at lower temperatures. Between 300 and 100 K, the emission intensity gradually increased, suggesting a significant reduction in energy loss due to nonradiative decay in this temperature range (Figure 2d). Meanwhile, the temperature-independent emission intensity observed below 100 K indicates cluster-centered emission properties controlled by cuprophilic interactions. Therefore, this investigation deduces that the emission of this NC originates from the synergistic interaction between a cluster-centered triplet excited state and metal–ligand charge transfer.

**Figure 2:**
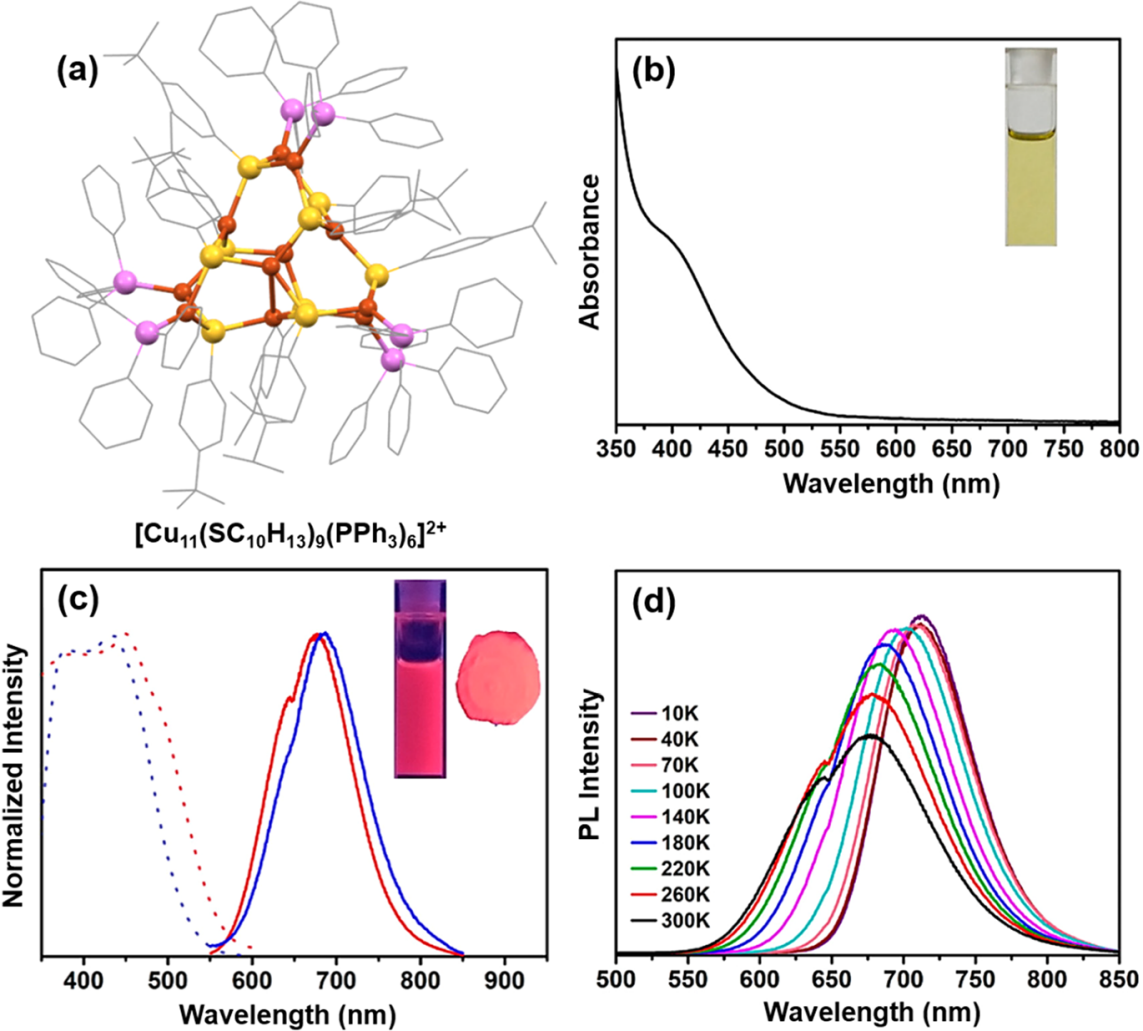
(a) Structural architecture of [Cu_11_(SC_10_H_13_)_9_(PPh_3_)_6_]^2+^ NC, (b) its absorbance in chloroform medium, (c) excitation (dotted line) and emission spectra (solid line) of this NC in solid state (red in color) and solution (blue in color), and (d) temperature dependence of emission in the solid state. Adapted with permission from ref (71). Copyright 2020 American Chemical Society.

Nematulloev et al. conducted the synthesis of the [Cu_15_(PPh_3_)_6_(PET)_13_]^2+^ (PET: 2-phenylethanthiolate) NC, employing another thiolate ligand.^72^ In the course of the reaction, ligand precursors were initially treated with [Cu(CH_3_CN)_4_]BF_4_ in an acetonitrile and chloroform solvent mixture, followed by NaBH_4_ reduction. Despite no apparent reduction occurring in the Cu(I) center, reduction remains a crucial step, vital for attaining a precise structural architecture. This NC features a distorted trigonal antiprismatic Cu_6_ core where cuprophilic interactions serve as the primary binding force. The detailed analysis of the Cu–Cu distances within this specific configuration unveils a range spanning from 2.56 to 2.93 Å. The overall structural arrangement embraces a distinct “triblade fan” like arrangement that imparts practical chirality to this NC. Notably, weak intermolecular ligand interactions, including π–π stacking and C–Hπ interactions, significantly contribute to the formation of centrosymmetric supramolecular dimers, involving the two opposite enantiomers. Anyway, in a chloroform medium, this NC displays a UV–vis absorbance at 404 nm. They identified that the primary localization of the estimated HOMO is within the Cu, P, and S atoms, while the LUMO extends across the central core and partial ligands. Upon excitation at 473 nm, it exhibits a PL emission at 720 nm. A comparative analysis of the PL emission intensity between the crystalline and solution states underscores significantly stronger emission in the crystalline state (QY 3.2%) compared to the weak emission observed in solution (QY 0.1%). The increased emission intensity observed in the solid state can be ascribed to the existence of extended π–π and C–Hπ intermolecular interactions, whereas the disordered configuration of cluster molecules in solvent hinders establishment of consistent intermolecular interactions, leading to a reduced emission intensity in the solution state. Moreover, a transition in the emission lifetime is noted, shifting from the nanosecond to the microsecond scale. This transition reveals the radiative recombination process for phosphorescence originating from an excited triplet state. Once again, the significance of intermolecular interaction has been demonstrated, with Jin et al. highlighting the favorable role of hydrogen bonding in the generation of red emission at 680 nm within a chiral arrangement of the [Cu_5_(S^t^Bu)_6_]^−^ NCs.^73^ In line with an earlier report, it was noted that the origin of this emission stems from an excited triplet state, primarily attributed to ligand-to-metal charge transfer. Lin et al. recently synthesized the 2-chloro-4-fluorobenzenethiol and phenyl phosphine-protected [Cu_13_H_10_(SC_6_H_3_ClF)_3_(PPh_3_)_7_] NC (Figure 3a) using a solvent-mediated precipitating synthesis method.^74^ In this intricate procedure, CuBr was initially stirred in an acetonitrile medium followed by the addition of methanol and dichloromethane with subsequent stirring. PPh_3_ and 2-chloro-4-fluorobenzenethiol were then introduced initially, and then the reduction was accomplished by adding NaBH_4_, which was confirmed as the source of hydrides in the reaction. The structural analysis reveals that the Cu_13_ kernel comprises four tetrahedrons that share three vertices, forming a distinct configuration resembling a triblade fan as previously noted. The Cu–Cu bond lengths in this configuration vary from 2.415 to 2.792 Å, suggesting more robust Cu–Cu interactions when compared to previously discussed Cu NCs. The steady-state UV–vis absorption spectrum exhibited four broad absorbance peaks at 305, 325, 340, and 360 nm (Figure 3b). The Kohn–Sham molecular orbital analysis was instrumental in elucidating the origins of these absorption peaks, unveiling transitions between the 3d and 4p orbitals of Cu. However, the remaining peaks were attributed to transitions between Cu (3d) and ligand π*. The excitation spectrum displayed three notable peaks, accompanied by a shoulder around 360 nm (Figure 3c). Excitation at 375 nm resulted in an emission spectrum with a maximum at 434 nm. However, at lower temperatures, there was an increase in the emission intensity, suggesting the potential occurrence of emission arising from the ligand-to-metal charge transfer process. So, the emission of this NC is mainly driven by the exchange of charges between surface ligands, which possess electron-donating capabilities, and the Cu atoms at the core, which have electron-withdrawing capabilities. In our attempt, we successfully synthesized the [Cu_58_(SC_3_H_7_)_36_(PPh_3_)_8_]^2+^ NC by using propanethiol.^75^ The reaction involves treating Cu(CH_3_CN)_4_BF_4_ with ligand precursors. This procedure occurs in a meticulously designed solvent mixture of acetonitrile and chloroform (4:1) at room temperature, followed by a crucial NaBH_4_ reduction step in methanol. The utilization of the ternary solvent medium plays a pivotal role in both regulating the reduction process and influencing the crystal growth. Upon a detailed structural analysis, we discovered that this NC exhibits a nested Keplerian architecture. At its core lies a cubic Cu_8_ geometry, surrounded by four cationic shells and five anionic shells. Notably, the average Cu–Cu distance within the cubic core measures 2.6541 Å, falling within the category of strong cuprophilic interactions. In the cationic shells, although the interaction is slightly less, it remains within the limits of the cuprophilic interactions. Among the five anionic shells, two are composed of thiolate ligands, one of phosphonate ligands, and the remaining two of hydrides. This NC exhibits a gradual decrease in absorbance from 250 nm onward without any prominent peaks. However, upon excitation at 405 nm, NC demonstrates an emission profile with a peak at 673 nm. The QY of this emission is measured at 0.38, and the emission lifetime is found to be 1.42 μs. Theoretical calculations unveiled that the HOMO of this NC is located in the −1.5263 eV band region with a triple degeneracy and a predominant composition of thiolate and hydride ligands. In contrast, the LUMO at −0.0045 eV is primarily derived from the Cu_8_ cubic core. Therefore, the observed emission is ascribed to electronic transitions between the combined shell and core. Interestingly, an analogous structure, [Cu_58_H_20_(PET)_36_(PPh_3_)_4_]^2+^, reported by Dong et al., shares a similar UV–vis absorption profile.^76^ However, they did not explore its luminescent properties. However, interestingly when one Cu atom was strategically removed from the cluster unit, generating [Cu_57_H_20_(PET)_36_(PPh_3_)_4_]^+^, the UV–vis absorption profile remained similar. Nonetheless, distinct differences in transient absorption properties were identified, emphasizing the impact of changes in metallic architecture on the generation of new phenomena during shell-to-core-charge transition processes.

**Figure 3:**
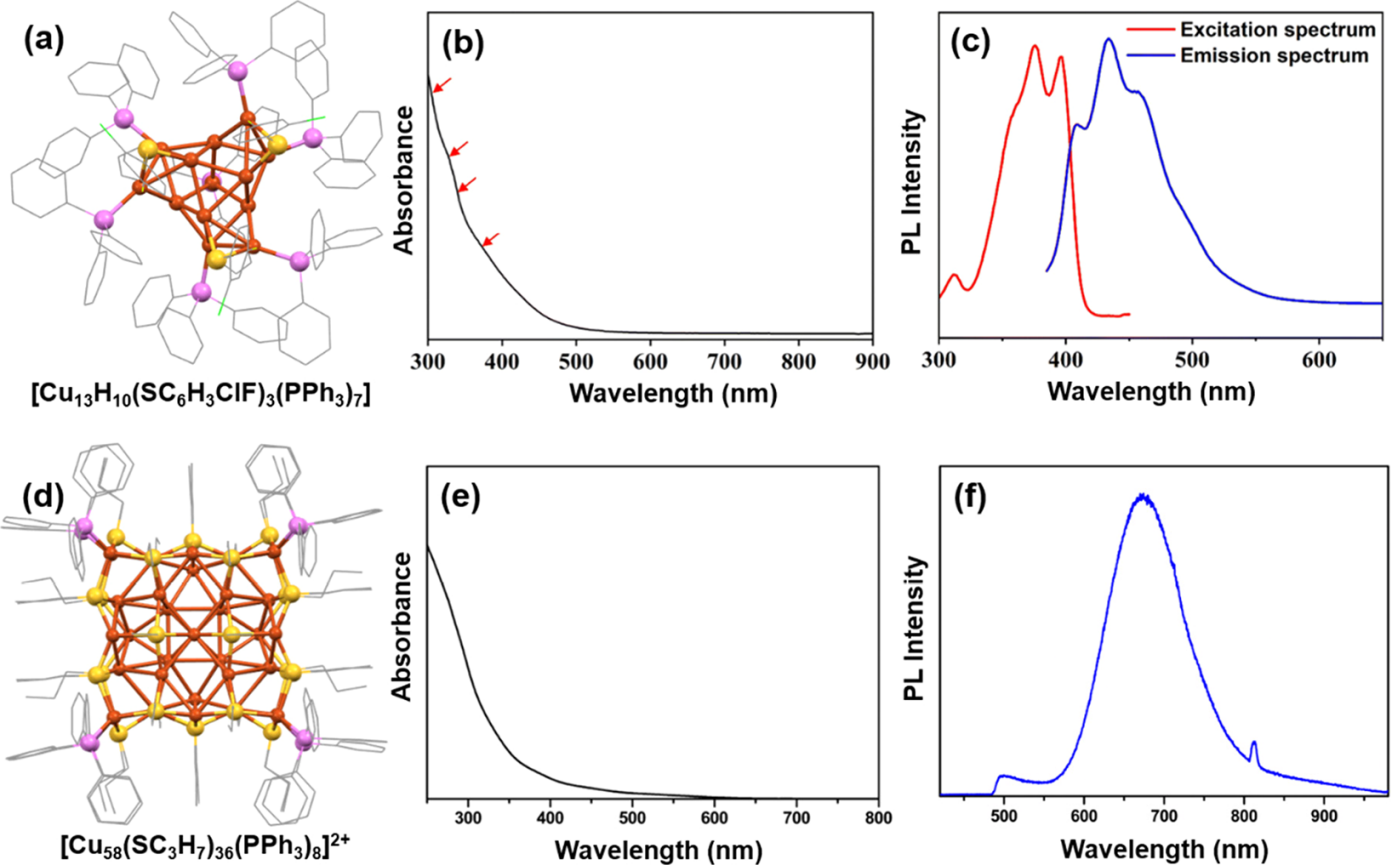
(a) Structural architecture of [Cu_13_H_10_(SC_6_H_3_ClF)_3_(PPh_3_)_7_] NC, (b) its absorbance in solution, and (c) excitation and emission in solution. Adapted with permission from ref (74). Copyright 2023 Royal Society of Chemistry. (d) Structural architecture of the [Cu_58_(SC_3_H_7_)_36_(PPh_3_)_8_]^2+^, (e) its absorbance in solution, and (f) its emission in chloroform medium. Adapted with permission from ref (75). Copyright 2023 Royal Society of Chemistry.

Thus, the variation in the number of Cu atoms in the cluster node results in distinct emission properties, each stemming from the unique characteristics of the cluster node. However, a commonality in all of the aforementioned examples is that all Cu atoms are monovalent. Given that Cu can exhibit variable oxidation states, exploring the emission properties in scenarios in which mixed-valence Cu atoms are present in the structure becomes highly desirable. Das et al. reported a core–shell Cu NC denoted by [Cu_18_H_3_(SC_10_H_15_)_12_(PPh_3_)_4_Cl_2_] NC (Figure 4a) where both Cu(0) and Cu(I) are present.^77^ The previously discussed ternary solvent reaction method was employed for the synthesis of this NC. Notably, their investigation unveiled that the structure is composed of a Cu_10_H_3_Cl_2_ core and a Cu_8_S_12_P_4_ shell. The distinctive construction of the core involves the fusion of a Cu_6_ octahedron and a Cu_5_ square pyramid through a vertex-sharing Cu(0) atom. The Cu–Cu distances within the core ranged from 2.475 to 2.989 Å, suggesting the presence of potential cuprophilic interactions. Furthermore, they also identified multiple point defects relative to the pseudoisostructural [Ag_23_(SC_2_H_4_Ph)_18_(PPh_3_)_8_] NC, contributing to structural stability through shell atom reconstruction.^78^ In a chloroform medium, this NC displays an absorbance spectrum featuring peaks at 390 and 450 nm (Figure 4b). These peaks are associated with transitions HOMO → LUMO+16 and HOMO → LUMO+8, respectively. Kohn–Sham molecular orbital analysis revealed significant d-orbital contributions to the occupied orbitals of the Cu_6_ octahedron, including contributions from the Cu_5_ square pyramid and nonbonding ligand states in the unoccupied orbitals, attributing the optical transitions between the two cores through Cu(0) center. Hence, the presence of the Cu(0) center emerges as a crucial factor in understanding the electronic transitions of the system. However, at 350 nm excitation, it exhibits violet emission with a peak at 420 nm, attributed to interband electronic relaxation within the core (QY of 0.32%, Figure 4c), with a lifetime of 0.26 ns. To enhance emission QY, the surface was rigidified through supramolecular adduct formation with β-cyclodextrin (β-CD), leading to a substantial improvement in the emission lifetime (Figure 4c). The identified reduction in nonradiative relaxation rates after adduct formation closely mirrors the suppression of intramolecular motion within the NC. So, the observed enhancement in emission behavior was attributed to the effective suppression of nonradiative relaxation rates. Emphasizing the importance of supramolecular adduct formation, this study underscored its role in enhancing both emission QY and lifetime, especially in solid-state emission properties where the formation of adduct restricts intramolecular motion. In another report, Wu et al. unveiled the defect-induced emission properties of Cu NCs.^79^ Dodecanethiol-capped Cu NCs were synthesized, and nanosheets were prepared by introducing dibenzyl ether. To enhance the metal defects on the surface, they added ethanol intentionally, which ultimately increased the Cu(I)-to-Cu(0) ratio. This specific composition notably impacts ligand-to-metal–metal charge transfer processes, consequently enhancing the radiative relaxation of excitons. Furthermore, Li et al. synthesized another Cu(0) containing NC, Cu_14_(C_2_B_10_H_10_S_2_)_6_(CH_3_CN)_8_, by treating Cu(CF_3_COO)_2_ with 1,2-dithiol-*o*-carborane in an acetonitrile-tetrahydrofuran medium.^80^ The structural analysis revealed that this NC contains a cubic Cu_14_ core, where each face is capped by bidentate carborane ligands and each vertex is capped by CH_3_CN. A noteworthy observation is that specific Cu–Cu distances within the core are shorter than typical metallic Cu–Cu bonds, indicating the presence of a Cu(0) state. The UV–vis absorbance peaks at 294 and 339 nm correspond to electronic transitions from HOMO–20 to LUMO and HOMO–6 to LUMO+1, respectively. Upon excitation at 400 nm, it emits at 637 and 661 nm (QY = 0.31), with a large lifetime of 5.13 μs, indicative of spin-forbidden triplet phosphorescence. The emission arises from transitions involving S-type HOMO–6 and ligand-based HOMOs to P-type LUMOs. Photoexcitation leads to electron influx into superatomic 1P orbitals, causing distortions in excited states, contributing to a substantial Stokes shift.

**Figure 4:**
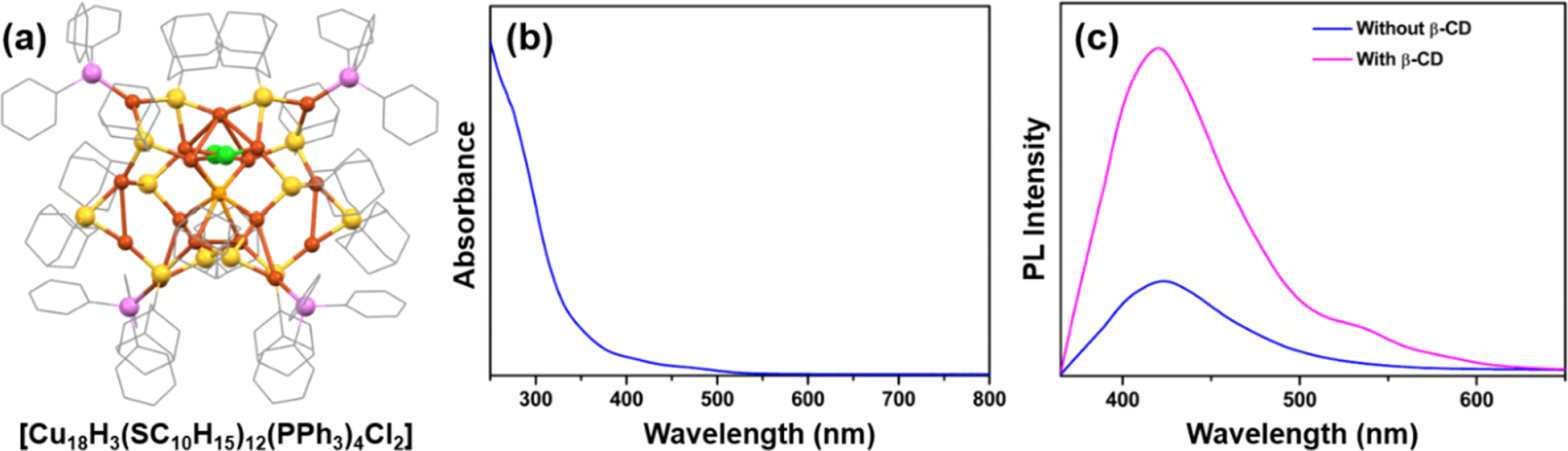
(a) Structural architecture of [Cu_18_H_3_(SC_10_H_15_)_12_(PPh_3_)_4_Cl_2_] NC, (b) its absorance in solution, and (c) its emission in solution with or without adduct of β-CD. Adapted with permission from ref (77). Copyright 2022 Royal Society of Chemistry.

The examples provided above clearly illustrate how the thiolate ligand, both independently and in conjunction with the phosphonate, plays a crucial role in controlling the emission properties of Cu NCs. Now, our attention turns toward understanding the emission characteristics of Cu NCs when an entirely different ligand system is introduced. Zhuo et al. synthesized [Cu_15_(^*t*^BuC≡C)_10_(CF_3_COO)_5_][^*t*^BuC≡CH] and [Cu_16_(^*t*^BuC≡C)_12_(CF_3_COO)_4_(CH_3_OH)_2_] NCs through an in situ comproportionation reaction, showcasing their distinctive thermochromic luminescence.^81^ During the reaction, Cu(CF_3_COO)_2_ reacts with ^*t*^BuC≡CH in methanol, facilitated by metallic copper powder. The cluster cores are linked through moderate cuprophilic interactions, with a slightly stronger connection observed in the Cu_15_ core. The Cu_15_ core-containing NC displays a broad absorption spectrum spanning from 230 to 500 nm, lacking a distinct identifiable peak. In contrast, the Cu_16_ core-containing NC reveals three well-defined peaks centered at 252, 330, and 450 nm within the same solid-state absorption region. The analysis attributes the peaks in the 250–400 nm and visible regions to the π → π* transition and charge transfer, respectively. Upon excitation at 365 nm, Cu_15_ and Cu_16_ core-containing NCs exhibit emission bands at 710 and 680 nm, respectively. The proposed association of these identified near-infrared emission bands is with a spin-forbidden excited state combined with cluster-centered characteristics influenced by cuprophilic interactions. Distinctive thermochromic luminescent behaviors were also noted, with the Cu_15_ core-containing NC showing the most pronounced response. Gradual cooling to 93 K results in a remarkable 17-fold enrichment in the emission intensity of that NC, accompanied by a progressive red shift in the peak from 710 to 793 nm. This improved emission intensity upon cooling is attributed to the reduction of the nonradiative decay. Whereas, the red-shifted phosphorescence is correlated with a decrease in the energy gap of the transition state, caused by shorter Cu–Cu contacts. They also identified a 1.7% reduction in the mean Cu–Cu distance from 273 to 93 K. So, the improved overlap of s and p atomic orbitals between neighboring Cu(I) centers reduces the energy gap of the cluster center transition state, resulting in emission at a longer wavelength. Zhang et al. discovered two additional Cu NCs that demonstrate near-infrared (NIR) emission.^82^ The synthesized [Cu_15_(^*t*^BuCC)_14_NO_3_] displays emission maximum at 871 nm when excited at 500 nm, while upon similar excitation, the [Cu_28_(^*t*^BuCC)_22_(SO_4_)_2_(OMe)_2_] NC exhibits emission maximum at 748 nm. This discrepancy is attributed to variations in their Cu–Cu distances, which, in turn, influence the mechanistic pathway of the emission. Jia et al. synthesized [Cu_31_(4-MeO-PhC≡C)_21_(dppe)_3_](ClO_4_)_2_ (dppe: 1,2-bis(diphenylphosphino)ethane) which exhibits an exceptional emission maximum at 1250 nm upon excitation at 480 nm.^83^ In an another study, Zhang et al. reported the circularly polarized luminescence of [Cu_14_(R/S-DPM)_8_](PF_6_)_6_ (DPM: 2-diphenyl-2-hydroxylmethylpyrrolidine-1-propyne) (R/S–Cu_14_) NCs (Figure 5a).^84^ The reaction involves the interaction between the chiral ligand and [Cu(MeCN)_4_]PF_6_ in dichloromethane. They detected an absorption peak at 348 nm accompanied by an extended shoulder at 430 nm, corresponding to HOMO–11 → LUMO and HOMO → LUMO+1 electronic transitions. These transitions involve orbitals composed of copper atoms and alkynyl groups. Remarkably, the solutions of these NCs did not emit but exhibited unique symmetric circular dichroism signals, distinct from the ligands. In contrast, in the crystalline state, R-Cu_14_ NCs displayed robust red emission with a notable QY of 0.082 (Figure 5b). They noted that the circularly polarized luminescence (CPL) response in the solid state aligned with the PL emission peak centered at 726 nm (Figure 5c). The considerably large lifetime of R-Cu_14_ (61.15 μs) and its Stokes shift suggested that the emission in the excited state had a triplet origin. Consequently, the red emission was proposed to stem from states that encompassed significant ligand-to-metal charge transfer mixed with a metal-centered *n*d^9^ (*n+*1) s^1^ state.^85,86^ So, the disparity in the emission and CPL between the solution and crystalline states of these NCs is attributed to their rigid structural conformation. Transitioning to the Cu chalcogenide cluster, Eichhöfer et al. conducted an extensive investigation into the electronic structure of eight distinct Cu chalcogenide clusters. These clusters encompass [Cu_12_S_6_(Ph_2_P(CH_2_)_5_PPh_2_)_4_] (1), [Cu_12_Se_6_(Ph_2_P(CH_2_)_8_PPh_2_)_4_] (2), [Cu_12_S_6_(Ph_2_PCpFeCpPPh_2_)_4_] (3), [Cu_12_S_6_(PPh_2_Et)_8_] (Et: ethyl) (4), [Cu_12_S_6_(PEt_3_)_8_] (5), [Cu_24_S_12_(PEt_2_Ph)_12_] (6), [Cu_20_S_10_(PPh_3_)_8_] (7), and [Cu_20_S_10_(P^*t*^Bu_3_)_8_] (8).^87^ They noticed that electronic transitions in all of these nanocrystals occur in the lower energy range. However, when the energies exceeded 2.5 eV, the dominant electron transition was from the orbitals within the cluster core to the ligand orbitals. This observation suggests a keen sensitivity to the structural arrangement of ligands. Specifically, they identified that this phenomenon is particularly linked to the presence of phenyl and phosphine ligand spheres. Concerning PL emission in the solid state, NCs 1, 2, 4, and 5 exhibited vibrant red emissions centered at ∼615–700 nm, decaying within a few microseconds, with the QY ranging from 21 to 63%. However, these emission intensities were temperature-dependent, reaching a QY of 100% below 100 K. Interestingly, they observed that the emission lifetimes of these NCs did not linearly correlate with the emission efficiency. Thus, they concluded that despite similar core architecture, the emission properties of these NCs significantly depended on their ligand structures and crystal packing. Moreover, NC 3 exhibited minimal red emission, even at low temperatures. The primary factor contributing to the reduction in emission intensity is the presence of ferrocenyl groups, which distribute the charge over both the ligands and the cluster core. While the dimeric core of NC 6 displayed PL emission at ∼680 nm, akin to the previously mentioned monomeric nanocrystals, notable variations in the atom–atom distances were observed. This suggests the resilience of the electronic properties of the core against substantial geometric distortions. Conversely, NC 7 manifested a broad emission at ∼820 nm, while NC 8 showed a comparatively weak emission at ∼575 nm. These variations can be attributed to their distinct structural architectures and changes in the core geometry. This comprehensive study provides valuable insights into the intricate electronic and photophysical properties of these Cu chalcogenide clusters, enhancing our understanding of their structural architecture.

**Figure 5:**
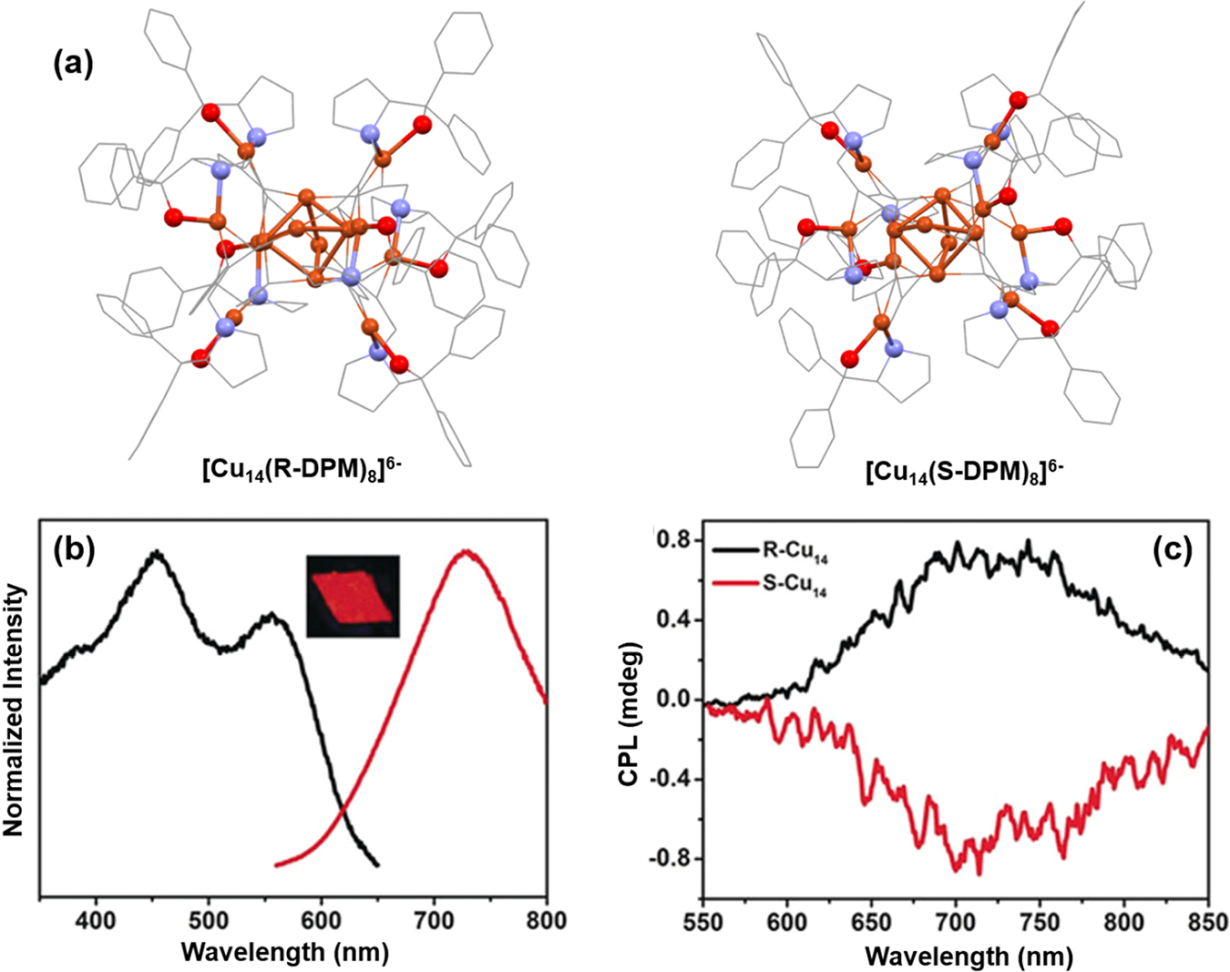
Structural architecture of (a) [Cu_14_(R-DPM)_8_]^6–^, (b) [Cu_14_(S-DPM)_8_]^6–^, (c) normalized excitation (black) and emission (red) of [Cu_14_(R-DPM)_8_]^6–^, and (c) CPL spectra of both enantiomers. Adapted with permission from ref (84). Copyright 2020 John Wiley and Sons.

Finally, it is worth noting that the emission properties of Cu NCs are governed by a complex interplay of various factors, each contributing to the determination of mechanistic pathways for emission generation and influencing electron transitions. Notably, certain factors continue to exert their influence on the emission properties even postsynthesis of the NCs. The intricacies involved in understanding which parameters are essential for modulating the emission properties of Cu NCs render this field challenging. However, the comprehensive discussion presented herein serves as a logical guide, paving the way for future research endeavors (Table 1). Examining the current state of research, the exploration of novel Cu NCs promises to enhance our comprehension by yielding new insights and solutions, particularly addressing issues such as a lower QY and emission stability. The emerging field of defect-induced structural architecture design, missing centered atom, and dangling bonds unveils intermediate energy levels via energy and/or charge transfer. This occurrence markedly influences the photophysical relaxation process, affecting exciton separation and recombination. Therefore, we anticipate that defect-induced emission studies will represent a pivotal advancement in this research domain. Furthermore, while aggregation-induced emission properties have surfaced in some instances, there remains ample unexplored territory, with both newly synthesized Cu NCs and existing ones. Although not discussed in detail here, the recent surge in alloying Cu NCs presents a compelling avenue with the potential to directly influence the electronic architecture of the NCs. As research progresses, it is anticipated that these diverse avenues will contribute to a more nuanced understanding of Cu NC emission properties, opening up new possibilities for technological applications and advancements in the field.

**Table 1:** Luminescence of Various Structurally Characterized Cu NCs

Cu NCs	Average Cu–Cu bond length (Å)	Excitation wavelength (nm)	Emission maximum (nm)	Reference
[Cu_7_(*p*-S-C_6_H_4_-NMe_2_)_7_(PPh_3_)_4_]	2.7871	400	560	(66)
Cu_7_(*p*-S-C_6_H_4_-OSiMe_3_)(SPh)_6_(PPh_3_)_4_]	2.8201	400	530	(66)
[Cu_8_(^***t***^BuC_6_H_4_S)_8_(PPh_3_)_4_]	2.9109	340	530	(68)
[Cu_8_(SPh)_8_(PPh_3_)_4_]	2.7452	405	655	(70)
[Cu_11_(SC_10_H_13_)_9_(PPh_3_)_6_]^2+^	3.0392	400	685	(71)
[Cu_13_H_10_(SC_6_H_3_ClF)_3_(PPh_3_)_7_]	2.6035	375	434	(74)
Cu_14_(C_2_B_10_H_10_S_2_)_6_(CH_3_CN)_8_	2.4926	400	637, 661	(80)
[Cu_14_(R/S-DPM)_8_](PF_6_)_6_	2.545	445	726	(84)
[Cu_15_(PPh_3_)_6_(PET)_13_]^2+^	2.7452	473	720	(72)
[Cu_15_(^***t***^BuC≡C)_10_(CF_3_COO)_5_][^***t***^BuC≡CH]	2.6331	365	710	(81)
[Cu_15_(^***t***^BuCC)_14_NO_3_]	2.7306	500	871	(82)
[Cu_16_(^***t***^BuC≡C)_12_(CF_3_COO)_4_(CH_3_OH)_2_]	2.6507	365	680	(81)
Cu_18_H(PhC_2_H_4_S)_14_(PPh_3_)_6_(NCS)_3_	3.29	-	688	(69)
[Cu_18_H_3_(SC_10_H_15_)_12_(PPh_3_)_4_Cl_2_]	2.7322	350	420	(77)
[Cu_28_(^***t***^BuCC)_22_(SO_4_)_2_(OMe)_2_]	2.6886	500	748	(82)
[Cu_31_(4-MeO-PhC≡C)_21_(dppe)_3_](ClO_4_)_2_	2.60	480	1250	(83)
[Cu_58_(SC_3_H_7_)_36_(PPh_3_)_8_]^2+^	2.6541	405	673	(75)

## References

[ref1] Jin R.; Zeng C.; Zhou M.; Chen Y. Atomically Precise Colloidal Metal Nanoclusters and Nanoparticles: Fundamentals and Opportunities. Chem. Rev. 2016, 116, 10346–10413. 10.1021/acs.chemrev.5b00703. 27585252

[ref2] Kang X.; Zhu M. Tailoring the Photoluminescence of Atomically Precise Nanoclusters. Chem. Soc. Rev. 2019, 48, 2422–2457. 10.1039/C8CS00800K. 30838373

[ref3] Shang L.; Dong S.; Nienhaus G. U. Ultra-small Fluorescent Metal Nanoclusters: Synthesis and Biological Applications. Nano Today 2011, 6, 401–418. 10.1016/j.nantod.2011.06.004.

[ref4] Biswas S.; Das A. K.; Mandal S. Surface Engineering of Atomically Precise M(I) Nanoclusters: From Structural Control to Room Temperature Photoluminescence Enhancement. Acc. Chem. Res. 2023, 56, 1838–1849. 10.1021/acs.accounts.3c00176. 37357739

[ref5] Du Y.; Sheng H.; Astruc D.; Zhu M. Atomically Precise Noble Metal Nanoclusters as Efficient Catalysts: a Bridge between Structure and Properties. Chem. Rev. 2020, 120, 526–622. 10.1021/acs.chemrev.8b00726. 30901198

[ref6] Hirai H.; Ito S.; Takano S.; Koyasu K.; Tsukuda T. Ligand-protected Gold/Silver Superatoms: Current Status and Emerging Trends. Chem. Sci. 2020, 11, 12233–12248. 10.1039/D0SC04100A. 34094434 PMC8162828

[ref7] Niihori Y.; Miyajima S.; Ikeda A.; Kosaka T.; Negishi Y. Vertex-Shared Linear Superatomic Molecules: Stepping Stones to Novel Materials Composed of Noble Metal Clusters. Small Sci. 2023, 3, 2300024 10.1002/smsc.202300024. PMC11935948 40213321

[ref8] Kurashige W.; Niihori Y.; Sharma S.; Negishi Y. Precise Synthesis, Functionalization and Application of Thiolate-Protected Gold Clusters. Coord. Chem. Rev. 2016, 320, 238–250. 10.1016/j.ccr.2016.02.013.

[ref9] Jin Y.; Zhang C.; Dong X.-Y.; Zang S.-Q.; Mak T. -C. -W. Shell Engineering to Achieve Modification and Assembly of Atomically-Precise Silver Clusters. Chem. Soc. Rev. 2021, 50, 2297–2319. 10.1039/D0CS01393E. 33443527

[ref10] Biswas S.; Das S.; Negishi Y. Progress and Prospects in the Design of Functional Atomically-precise Ag (I)-thiolate Nanoclusters and Their Assembly Approaches. Coord. Chem. Rev. 2023, 492, 215255 10.1016/j.ccr.2023.215255.

[ref11] Biswas S.; Das S.; Negishi Y. Advances in Cu Nanocluster Catalyst Design: Recent Progress and Promising Applications. Nanoscale Horiz 2023, 8, 1509–1522. 10.1039/D3NH00336A. 37772632

[ref12] Liu Y.; Yu J.; Lun Y.; Wang Y.; Wang Y.; Song S. Ligand Design in Atomically Precise Copper Nanoclusters and Their Application in Electrocatalytic Reactions. Adv. Funct. Mater. 2023, 33, 2304184 10.1002/adfm.202304184.

[ref13] Liu X.; Astruc D. Atomically Precise Copper Nanoclusters and Their Applications. Coord. Chem. Rev. 2018, 359, 112–126. 10.1016/j.ccr.2018.01.001.

[ref14] Han Z.; Dong X.-Y.; Luo P.; Li S.; Wang Z.-Y.; Zang S.-Q.; Mak T. -C. -W. Ultrastable Atomically Precise Chiral Silver Clusters with more than 95% Quantum Efficiency. Sci. Adv. 2020, 6, eaay0107 10.1126/sciadv.aay0107. 32083176 PMC7007243

[ref15] Dhayal R. S.; van Zyl W. E.; Liu C. Polyhydrido Copper Clusters: Synthetic Advances, Structural Diversity, and Nanocluster-to-Nanoparticle Conversion. Acc. Chem. Res. 2016, 49, 86–95. 10.1021/acs.accounts.5b00375. 26696469

[ref16] Wu Q. J.; Si D. H.; Sun P.-P.; Dong Y.-L.; Zheng S.; Chen Q.; Ye S. H.; Sun D.; Cao R.; Huang Y.-B. Atomically Precise Copper Nanoclusters for Highly Efficient Electroreduction of CO2 towards Hydrocarbons via Breaking the Coordination Symmetry of Cu Site. Angew. Chem., Int. Ed. 2023, 62, e202306822 10.1002/anie.202306822. 37468435

[ref17] Luo G.-G.; Pan Z.-H.; Han B.-L.; Dong G.-L.; Deng C.-L.; Azam M.; Tao Y.-W.; He J.; Sun C.-F.; Sun D. Total Structure, Electronic Structure and Catalytic Hydrogenation Activity of Metal-Deficient Chiral Polyhydride Cu_57_ Nanoclusters. Angew. Chem., Int. Ed. 2023, 62, e202306849 10.1002/anie.202306849. 37469101

[ref18] Baghdasaryan A.; Bürgi T. Copper Nanoclusters: Designed Synthesis, Structural Diversity, and Multiplatform Applications. Nanoscale 2021, 13, 6283–6340. 10.1039/D0NR08489A. 33885518

[ref19] Wang Z.; Chen B.; Rogach A. L. Synthesis, Optical Properties and Applications of Light-emitting Copper Nanoclusters. Nanoscale Horiz 2017, 2, 135–146. 10.1039/C7NH00013H. 32260657

[ref20] Chakraborty I.; Pradeep T. Atomically Precise Clusters of Noble Metals: Emerging Link between Atoms and Nanoparticles. Chem. Rev. 2017, 117, 8208–8271. 10.1021/acs.chemrev.6b00769. 28586213

[ref21] Shahsavari S.; Hadian-Ghazvini S.; Saboor F. H.; Oskouie I. M.; Hasany M.; Simchi A.; Rogach A. L. Ligand Functionalized Copper Nanoclusters for Versatile Applications in Catalysis, Sensing, Bioimaging, and Optoelectronics. Mater. Chem. Front. 2019, 3, 2326–2356. 10.1039/C9QM00492K.

[ref22] Kolay S.; Bain D.; Maity S.; Devi A.; Patra A.; Antoine R. Self-assembled Metal Nanoclusters: Driving Forces and Structural Correlation with Optical Properties. Nanomaterials 2022, 12, 544 10.3390/nano12030544. 35159891 PMC8838213

[ref23] Yan J.; Teo B. K.; Zheng N. Surface Chemistry of Atomically Precise Coinage–Metal Nanoclusters: From Structural Control to Surface Reactivity and Catalysis. Acc. Chem. Res. 2018, 51, 3084–3093. 10.1021/acs.accounts.8b00371. 30433756

[ref24] Zeng C.; Chen Y.; Das A.; Jin R. Transformation Chemistry of Gold Nanoclusters: from One Stable Size to Another. J. Phys. Chem. Lett. 2015, 6, 2976–2986. 10.1021/acs.jpclett.5b01150. 26267191

[ref25] Gratious S.; Mukherjee S.; Mandal S. Co-reactant-Free Transformation in Atomically Precise Metal Nanoclusters. J. Phys. Chem. Lett. 2022, 13, 9014–9027. 10.1021/acs.jpclett.2c02330. 36149644

[ref26] Han B.-L.; Liu Z.; Feng L.; Wang Z.; Gupta R. K.; Aikens C. M.; Tung C.-H.; Sun D. Polymorphism in Atomically Precise Cu_23_ Nanocluster Incorporating Tetrahedral [Cu_4_]0 Kernel. J. Am. Chem. Soc. 2020, 142 (12), 5834–5841. 10.1021/jacs.0c01053. 32126754

[ref27] Dhayal R. S.; Liao J.-H.; Lin Y.-R.; Liao P.-K.; Kahlal S.; Saillard J.-Y.; Liu C. A Nanospheric Polyhydrido Copper Cluster of Elongated Triangular Orthobicupola Array: Liberation of H_2_ from Solar Energy. J. Am. Chem. Soc. 2013, 135, 4704–4707. 10.1021/ja401576s. 23472670

[ref28] Yuan P.; Chen R.; Zhang X.; Chen F.; Yan J.; Sun C.; Ou D.; Peng J.; Lin S.; Tang Z.; et al. Ether-Soluble Cu_53_ Nanoclusters as an Effective Precursor of High-quality CuI Films for Optoelectronic Applications. Angew. Chem., Int. Ed. 2019, 58, 835–839. 10.1002/anie.201812236. 30406951

[ref29] Matus M. F.; Häkkinen H. Understanding Ligand-Protected Noble Metal Nanoclusters at Work. Nat. Rev. Mater. 2023, 8, 372 10.1038/s41578-023-00537-1.

[ref30] Sahoo K.; Gazi T. R.; Roy S.; Chakraborty I. Nanohybrids of Atomically Precise Metal Nanoclusters. Commun. Chem. 2023, 6, 157 10.1038/s42004-023-00958-7. 37495665 PMC10372104

[ref31] Bera D.; Goswami N. Driving Forces and Routes for Aggregation-induced Emission-based Highly Luminescent Metal Nanocluster Assembly. J. Phys. Chem. Lett. 2021, 12, 9033–9046. 10.1021/acs.jpclett.1c02406. 34516135

[ref32] Joshi C. P.; Bootharaju M. S.; Bakr O. M. Tuning Properties in Silver Clusters. J. Phys. Chem. Lett. 2015, 6, 3023–3035. 10.1021/acs.jpclett.5b00934. 26267198

[ref33] Goswami N.; Yao Q.; Luo Z.; Li J.; Chen T.; Xie J. Luminescent Metal Nanoclusters with Aggregation-induced Emission. J. Phys. Chem. Lett. 2016, 7, 962–975. 10.1021/acs.jpclett.5b02765. 26912457

[ref34] Li B.; Fan H.-T.; Zang S.-Q.; Li H.-Y.; Wang L.-Y. Metal-containing Crystalline Luminescent Thermochromic Materials. Coord. Chem. Rev. 2018, 377, 307–329. 10.1016/j.ccr.2018.09.004.

[ref35] Lettieri M.; Palladino P.; Scarano S.; Minunni M. Copper Nanoclusters and Their Application for Innovative Fluorescent Detection Strategies: An Overview. Sens. Actuators Rep. 2022, 4, 100108 10.1016/j.snr.2022.100108.

[ref36] Qian S.; Wang Z.; Zuo Z.; Wang X.; Wang Q.; Yuan X. Engineering Luminescent Metal Nanoclusters for Sensing Applications. Coord. Chem. Rev. 2022, 451, 214268 10.1016/j.ccr.2021.214268.

[ref37] Yuan L.; Liang M.; Hummel M.; Shao C.; Lu S. Rational Design Copper Nanocluster-Based Fluorescent Sensors towards Heavy Metal Ions: A Review. Chemosensors 2023, 11 (3), 159 10.3390/chemosensors11030159.

[ref38] Jia X.; Li J.; Wang E. Cu Nanoclusters with Aggregation Induced Emission Enhancement. Small 2013, 9, 3873–3879. 10.1002/smll.201300896. 23670847

[ref39] Barthel M. J.; Angeloni I.; Petrelli A.; Avellini T.; Scarpellini A.; Bertoni G.; Armirotti A.; Moreels I.; Pellegrino T. Synthesis of Highly Fluorescent Copper Clusters Using Living Polymer Chains as Combined Reducing Agents and Ligands. ACS Nano 2015, 9, 11886–11897. 10.1021/acsnano.5b04270. 26512975

[ref40] Zhao M.; Sun L.; Crooks R. M. Preparation of Cu Nanoclusters within Dendrimer Templates. J. Am. Chem. Soc. 1998, 120, 4877–4878. 10.1021/ja980438n.

[ref41] Siwach O. P.; Sen P. Synthesis and Study of Fluorescence Properties of Cu Nanoparticles. J. Nanoparticle Res. 2008, 10, 107–114. 10.1007/s11051-008-9372-5.

[ref42] Vázquez-Vázquez C.; Banobre-Lopez M.; Mitra A.; Lopez-Quintela M. A.; Rivas J. Synthesis of Small Atomic Copper Clusters in Microemulsions. Langmuir 2009, 25, 8208–8216. 10.1021/la900100w. 19545135

[ref43] Yu H.; Rao B.; Jiang W.; Yang S.; Zhu M. The Photoluminescent Metal Nanoclusters with Atomic Precision. Coord. Chem. Rev. 2019, 378, 595–617. 10.1016/j.ccr.2017.12.005.

[ref44] Vilar-Vidal N.; Rivas J.; Lopez-Quintela M. A. Size Dependent Catalytic Activity of Reusable Subnanometer Copper (0) Clusters. ACS Catal. 2012, 2, 1693–1697. 10.1021/cs300355n.

[ref45] Goswami N.; Giri A.; Bootharaju M.; Xavier P. L.; Pradeep T.; Pal S. K. Copper Quantum Clusters in Protein Matrix: Potential Sensor of Pb^2+^ Ion. Anal. Chem. 2011, 83, 9676–9680. 10.1021/ac202610e. 22050123

[ref46] Zhao X. J.; Huang C. Z. Water-soluble Luminescent Copper Nanoclusters Reduced and Protected by Histidine for Sensing of Guanosine 5′-triphosphate. New J. Chem. 2014, 38, 3673–3677. 10.1039/C4NJ00731J.

[ref47] Sun D.; Yuan S.; Wang H.; Lu H.-F.; Feng S.-Y.; Sun D.-F. Luminescence Thermochromism of Two Entangled Copper-Iodide Networks with a Large Temperature-Dependent Emission Shift. Chem. Commun. 2013, 49, 6152–6154. 10.1039/c3cc42741b. 23728151

[ref48] Shan X.-c.; Jiang F.-l.; Yuan D.-q.; Zhang H.-b.; Wu M.-y.; Chen L.; Wei J.; Zhang S.-q.; Pan J.; Hong M.-c. A Multi-metal-cluster MOF with Cu_4_I_4_ and Cu_6_S_6_ as Functional Groups Exhibiting Dual Emission with Both Thermochromic and Near-IR Character. Chem. Sci. 2013, 4, 1484–1489. 10.1039/c3sc21995j.

[ref49] Yuan P.; He T.; Zhou Y.; Yin J.; Zhang H.; Zhang Y.; Yuan X.; Dong C.; Huang R.; Shao W.; et al. Hybrid Thermally Activated Nanocluster Fluorophores for X-ray Scintillators. ACS Energy Lett. 2023, 8, 5088–5097. 10.1021/acsenergylett.3c02050.

[ref50] Liu X.; Jiang Y.; Li F.; Xu X.; Li R.; Zhu W.; Ni J.; Ding C.; Liu S.; Zhao Q. Thermally Activated Delayed Fluorescent Scintillators Based on Mononuclear Copper (I) Halide Complexes for High-Resolution X-Ray Imaging. Adv. Optical Mater. 2023, 11, 2202169 10.1002/adom.202202169.

[ref51] Hu Q.; Zhang C.; Wu X.; Liang G.; Wang L.; Niu X.; Wang Z.; Si W. D.; Han Y.; Huang R.; et al. Highly Effective Hybrid Copper (I) Iodide Cluster Emitter with Negative Thermal Quenched Phosphorescence for X-Ray Imaging. Angew. Chem., Int. Ed. 2023, 62, e202217784 10.1002/anie.202217784. 36647290

[ref52] Zhao S. S.; Wang L.; Liu Y.; Chen L.; Xie Z. Stereochemically Dependent Synthesis of two Cu (I) Cluster-based Coordination Polymers with Thermochromic Luminescence. Inorg. Chem. 2017, 56, 13975–13981. 10.1021/acs.inorgchem.7b02123. 29099185

[ref53] Kim T. H.; Shin Y. W.; Jung J. H.; Kim J. S.; Kim J. Crystal-to-Crystal Transformation between Three CuI Coordination Polymers and Structural Evidence for Luminescence Thermochromism. Angew. Chem., Int. Ed. 2008, 47, 685–688. 10.1002/anie.200704349. 18080268

[ref54] Wang L.; Yan X.; Tian G.; Xie Z.; Shi S.; Zhang Y.; Li S.; Sun X.; Sun J.; He J.; et al. Chiral Copper-hydride Nanoclusters: Synthesis, Structure, and Assembly. Dalton Trans 2023, 52, 3371–3377. 10.1039/D2DT03788B. 36810425

[ref55] Zeng S.; Ge X.; Deng H.; Hao S.; Zhang Z.; Teo B. K.; Sun C. Synthesis and Structure of Polyhydrido Copper Nanocluster [Cu_14_H_10_(PPh_3_)_8_(SPhMe_2_)_3_]^+^: Symmetry-Breaking by Thiolate Ligands to form Racemic Pairs of Chiral Clusters in Solid-State. J. Cluster Sci. 2023, 1–5. 10.1007/s10876-023-02469-w.

[ref56] Dong G.; Pan Z.; Han B.; Tao Y.; Chen X.; Luo G.-G.; Sun P.; Sun C.; Sun D. Multi-layer 3D Chirality and Double-Helical Assembly in a Copper Nanocluster with a Triple-Helical Cu_15_ Core. Angew. Chem., Int. Ed. 2023, 62, e202302595 10.1002/anie.202302595. 37052323

[ref57] Sun C.; Mammen N.; Kaappa S.; Yuan P.; Deng G.; Zhao C.; Yan J.; Malola S.; Honkala K.; Häkkinen H. Atomically Precise, Thiolated Copper–hydride Nanoclusters as Single-site Hydrogenation Catalysts for Ketones in Mild Conditions. ACS Nano 2019, 13, 5975–5986. 10.1021/acsnano.9b02052. 31067029 PMC6750866

[ref58] Lee S.; Bootharaju M. S.; Deng G.; Malola S.; Baek W.; Hakkinen H.; Zheng N.; Hyeon T. [Cu_32_(PET)_24_H_8_Cl_2_](PPh_4_)_2_: A Copper Hydride Nanocluster with a Bisquare Antiprismatic Core. J. Am. Chem. Soc. 2020, 142, 13974–13981. 10.1021/jacs.0c06577. 32672452

[ref59] Dong C.; Huang R.-W.; Chen C.; Chen J.; Nematulloev S.; Guo X.; Ghosh A.; Alamer B.; Hedhili M. N.; Isimjan T.-T.; Han Y.; Mohammed O. F.; Bakr O. M. [Cu_36_H_10_(PET)_24_(PPh_3_)_6_Cl_2_] Reveals Surface Vacancy Defects in Ligand-Stabilized Metal Nanoclusters. J. Am. Chem. Soc. 2021, 143, 11026–11035. 10.1021/jacs.1c03402. 34255513

[ref60] Ghosh A.; Huang R.-W.; Alamer B.; Abou-Hamad E.; Hedhili M. N.; Mohammed O. F.; Bakr O. M. [Cu_61_(S^*t*^Bu)_26_S_6_Cl_6_H_14_]^+^: A Core–Shell Superatom Nanocluster with a Quasi-J 36 Cu19 Core and an “18-Crown-6” Metal-Sulfide-like Stabilizing Belt. ACS Mater. Lett. 2019, 1, 297–302. 10.1021/acsmaterialslett.9b00122.

[ref61] Tang J.; Liu C.; Zhu C.; Sun K.; Wang H.; Yin W.; Xu C.; Li Y.; Wang W.; Wang L.; Wu R.; Liu C.; Huang J. High-Nuclearity and Thiol Protected Core–shell [Cu_75_(S-Adm)_32_]^2+^: Distorted Octahedra Fixed to Cu_15_ Core via Strong Cuprophilic Interactions. Nanoscale 2023, 15, 2843–2848. 10.1039/D2NR05921E. 36688503

[ref62] Huang R.-W.; Yin J.; Dong C.; Ghosh A.; Alhilaly M. J.; Dong X.; Hedhili M. N.; Abou-Hamad E.; Alamer B.; Nematulloev S.; Hay Y.; Mohammed O. F.; Bakr O. M. [Cu_81_(PhS)_46_(^*t*^BuNH_2_)_10_(H)_32_]^3+^ Reveals the Coexistence of Large Planar Cores and Hemispherical Shells in High-nuclearity Copper Nanoclusters. J. Am. Chem. Soc. 2020, 142, 8696–8705. 10.1021/jacs.0c00541. 32315164

[ref63] Zhang L.-M.; Mak T. -C. -W. Comproportionation Synthesis of Copper (I) Alkynyl Complexes Encapsulating Polyoxomolybdate Templates: Bowl-shaped Cu_33_ and Peanut-shaped Cu_62_ Nanoclusters. J. Am. Chem. Soc. 2016, 138, 2909–2912. 10.1021/jacs.5b12103. 26899875

[ref64] Nguyen T.-A. D.; Jones Z. R.; Goldsmith B.-R.; Buratto W. R.; Wu G.; Scott S. L.; Hayton T. W. A Cu_25_ Nanocluster with Partial Cu (0) Character. J. Am. Chem. Soc. 2015, 137, 13319–13324. 10.1021/jacs.5b07574. 26422670

[ref65] Chakrahari K.-K.; Liao J.-H.; Kahlal S.; Liu Y.-C.; Chiang M.-H.; Saillard J.-Y.; Liu C. [Cu_13_{S_2_CNnBu_2_}_6_(acetylide)_4_]^+^: A Two-Electron Superatom. Angew. Chem., Int. Ed. 2016, 55, 14704–14708. 10.1002/anie.201608609. 27781357

[ref66] Langer R.; Yadav M.; Weinert B.; Fenske D.; Fuhr O. Luminescence in Functionalized Copper Thiolate Clusters–synthesis and Structural Effects. Eur. J. Inorg. Chem. 2013, 2013, 3623–3631. 10.1002/ejic.201300155.

[ref67] Cook A.-W.; Jones Z.-R.; Wu G.; Teat S.-J.; Scott S.-L.; Hayton T. W. Synthesis and Characterization of “Atlas-Sphere” Copper Nanoclusters: New Insights into the Reaction of Cu^2+^ with Thiols. Inorg. Chem. 2019, 58, 8739–8749. 10.1021/acs.inorgchem.9b01140. 31198031

[ref68] Sun P. P.; Han B. L.; Li H. G.; Zhang C. K.; Xin X.; Dou J. M.; Gao Z. Y.; Sun D. Real-Time Fluorescent Monitoring of Kinetically Controlled Supramolecular Self-Assembly of Atom-Precise Cu_8_ Nanocluster. Angew. Chem., Int. Ed. 2022, 61, e202200180 10.1002/anie.202200180. 35191142

[ref69] Dong G.; Pan Z.; Han B.; Tao Y.; Chen X.; Luo G.; Sun P.; Sun C.; Sun D. Multi-layer 3D Chirality and Double-Helical Assembly in a Copper Nanocluster with a Triple-Helical Cu_15_ Core. Angew. Chem., Int. Ed. 2023, 62, e202302595 10.1002/anie.202302595. 37052323

[ref70] Ke F.; Song Y.; Li H.; Zhou C.; Du Y.; Zhu M. Sub-nanometer Cu (I) Clusters: Coordination-Modulated (Se vs. S) Atom-packing Mode and Emission. Dalton Trans 2019, 48, 13921–13924. 10.1039/C9DT02908G. 31508627

[ref71] Li H.; Zhai H.; Zhou C.; Song Y.; Ke F.; Xu W.-W.; Zhu M. Atomically Precise Copper Cluster with Intensely Near-Infrared Luminescence and Its Mechanism. J. Phys. Chem. Lett. 2020, 11, 4891–4896. 10.1021/acs.jpclett.0c01358. 32490675

[ref72] Nematulloev S.; Huang R.-W.; Yin J.; Shkurenko A.; Dong C.; Ghosh A.; Alamer B.; Naphade R.; Hedhili M.-N.; Maity P.; Eddaoudi M.; Mohammed O. F.; Bakr O. M. [Cu_15_(PPh_3_)_6_(Pet)_13_]^2+^: A Copper Nanocluster with Crystallization Enhanced Photoluminescence. Small 2021, 17, 2006839 10.1002/smll.202006839. 33739606

[ref73] Jin Y.; Li S.; Han Z.; Yan B.-J.; Li H. Y.; Dong X.-Y.; Zang S.-Q. Cations Controlling the Chiral Assembly of Luminescent Atomically Precise Copper (I) Clusters. Angew. Chem., Int. Ed. 2019, 58, 12143 10.1002/anie.201906614. 31267660

[ref74] Lin X.; Tang J.; Zhu C.; Wang L.; Yang Y.; Wu R.-A.; Fan H.; Liu C.; Huang J. Solvent-mediated Precipitating Synthesis and Optical Properties of Polyhydrido Cu_13_ Nanoclusters with Four Vertex-sharing Tetrahedrons. Chem. Sci. 2023, 14, 994–1002. 10.1039/D2SC06099J. 36755712 PMC9890966

[ref75] Biswas S.; Hossian S.; Kosaka T.; Sakai J.; Arima D.; Niihori Y.; Mitsui M.; Jiang D.-e.; Das S.; Wang S.; Negishi Y. Nested Keplerian architecture of [Cu_58_H_20_(SPr)_36_(PPh_3_)_8_]^2+^ nanoclusters. Chem. Commun. 2023, 59, 9336–9339. 10.1039/D3CC01811C. 37404125

[ref76] Dong C.; Huang R.-W.; Sagadevan A.; Yuan P.; Gutiérrez-Arzaluz L.; Ghosh A.; Nematulloev S.; Alamer B.; Mohammed O. F.; Hussain I.; Rueping M.; Bakr O. M. Isostructural Nanocluster Manipulation Reveals Pivotal Role of One Surface Atom in Click Chemistry. Angew. Chem., Int. Ed. 2023, 62, e202307140 10.1002/anie.202307140. 37471684

[ref77] Das A. K.; Biswas S.; Wani V. S.; Nair A. S.; Pathak B.; Mandal S. [Cu_18_H_3_(S-Adm)_12_(PPh_3_)_4_Cl_2_]: Fusion of Platonic and Johnson Solids Through a Cu(0) Center and Its Photophysical Properties. Chem. Sci. 2022, 13, 7616–7625. 10.1039/D2SC02544B. 35872832 PMC9241973

[ref78] Liu C.; Li T.; Abroshan H.; Li Z.; Zhang C.; Kim H.-J.; Li G.; Jin R. Chiral Ag_23_ Nanocluster with Open Shell Electronic Structure and Helical Face-centered Cubic Framework. Nat. Commun. 2018, 9, 744 10.1038/s41467-018-03136-9. 29467372 PMC5821857

[ref79] Wu Z.; Liu H.; Li T.; Liu J.; Yin J.; Mohammed O. F.; Bakr O. M.; Liu Y.; Yang B.; Zhang H. Contribution of Metal Defects in the Assembly Induced Emission of Cu Nanoclusters. J. Am. Chem. Soc. 2017, 139, 4318–4321. 10.1021/jacs.7b00773. 28318238

[ref80] Li Y. L.; Wang J.; Luo P.; Ma X.-H.; Dong X. Y.; Wang Z.-Y.; Du C.-X.; Zang S.-Q.; Mak T. -C. -W. Cu_14_ Cluster with Partial Cu(0) Character: Difference in Electronic Structure from Isostructural Silver Analog. Adv. Sci. 2019, 6, 1900833 10.1002/advs.201900833. PMC6755520 31559130

[ref81] Zhuo H. Y.; Su H. F.; Cao Z. Z.; Liu W.; Wang S. A.; Feng L.; Zhuang G. L.; Lin S. C.; Kurmoo M.; Tung C. H.; et al. High-Nuclear Organometallic Copper (I)–Alkynide Clusters: Thermochromic Near-Infrared Luminescence and Solution Stability. Chem.—Eur. J. 2016, 22, 17619–17626. 10.1002/chem.201603797. 27730682

[ref82] Zhang L. L.-M.; Zhou G.; Zhou G.; Lee H.-K.; Zhao N.; Prezhdo O. V.; Mak T. -C. -W. Core-dependent Properties of Copper Nanoclusters: Valence-Pure Nanoclusters as NIR TADF Emitters and Mixed-Valence Ones as Semiconductors. Chem. Sci. 2019, 10, 10122–10128. 10.1039/C9SC03455B. 32055367 PMC7003970

[ref83] Jia T.; Guan Z.-J.; Zhang C.; Zhu X.-Z.; Chen Y.-X.; Zhang Q.; Yang Y.; Sun D. Eight-Electron Superatomic Cu_31_ Nanocluster with Chiral Kernel and NIR-II Emission. J. Am. Chem. Soc. 2023, 145, 10355–10363. 10.1021/jacs.3c02215. 37104621

[ref84] Zhang M. M.; Dong X. Y.; Wang Z. Y.; Li H. Y.; Li S. J.; Zhao X.; Zang S.-Q. AIE Triggers the Circularly Polarized Luminescence of Atomically Precise Enantiomeric Copper (I) Alkynyl Clusters. Angew. Chem., Int. Ed. 2020, 59, 10052 10.1002/anie.201908909. 31469491

[ref85] Lo W.-Y.; Lam C.-H.; Yam V. W.-W.; Zhu N.; Cheung K.-K.; Fathallah S.; Messaoudi S.; Le Guennic B.; Kahlal S.; Halet J.-F. Synthesis, Photophysics, Electrochemistry, Theoretical, and Transient Absorption Studies of Luminescent Copper (I) and Silver (I) Diynyl Complexes. X-ray Crystal Structures of [Cu_3_(μ-dppm)_3_(μ_3_-η^1^-C⋮CC⋮CPh)_2_]PF_6_ and [Cu_3_(μ-dppm)_3_(μ_3_-η_1_-C⋮CC⋮CH)_2_]PF_6_. J. Am. Chem. Soc. 2004, 126, 7300–7310. 10.1021/ja049300x. 15186167

[ref86] Yam V. W. W.; Fung W. K. M.; Cheung K. K. Synthesis, Structure, Photophysics, and Excited-State Redox Properties of the Novel Luminescent Tetranuclear Acetylidocopper (I) Complex [Cu_4_(μ-dppm)_4_(μ_4_-η^1^,η^2^-C≡C-)](BF_4_)_2_. Angew. Chem., Int. Ed. Engl. 1996, 35, 1100–1102. 10.1002/anie.199611001.

[ref87] Eichhöfer A.; Buth G.; Lebedkin S.; Kühn M.; Weigend F. Luminescence in Phosphine-Stabilized Copper Chalcogenide Cluster Molecules-A Comparative Study. Inorg. Chem. 2015, 54, 9413–9422. 10.1021/acs.inorgchem.5b01146. 26378617

